# Systematically Exploring the Antitumor Mechanisms of Core Chinese Herbs on Hepatocellular Carcinoma: A Computational Study

**DOI:** 10.1155/2020/2396569

**Published:** 2020-09-15

**Authors:** Zhulin Wu, Li He, Lianan Wang, Lisheng Peng

**Affiliations:** ^1^The Fourth Clinical Medical College of Guangzhou University of Chinese Medicine, Shenzhen 518033, China; ^2^Shenzhen Traditional Chinese Medicine Hospital, Shenzhen 518033, China

## Abstract

**Objective:**

Chinese herbs play a positive role in the management of hepatocellular carcinoma (HCC) in China. However, it is not clear which of Chinese herbs are critical for the treatment of HCC. Besides, mechanisms of CCHs in the treatment of HCC remain unclear. Hence, our goal is to identify the core Chinese herbs (CCHs) for treating HCC and explore their antitumor mechanism.

**Methods:**

Firstly, clinical traditional Chinese medicine (TCM) prescriptions for HCC were collected from Chinese National Knowledge Infrastructure (CNKI) database, and then, data mining software was used to identify CCHs. After that, bioactive compounds and corresponding target genes of CCHs were obtained using three TCM databases, and target genes of HCC were acquired from MalaCards and OMIM. Subsequently, common target genes of CCHs and HCC were screened. Moreover, biological functions and pathways were analyzed, and Cytoscape plugin cytoHubba was used to identify hub genes. Finally, prognostic values of hub genes were verified by survival analysis, and the molecular docking approach was utilized to validate the interactions between targets and bioactive compounds of CCHs.

**Results:**

Eight CCHs were determined from 630 prescriptions, and 100 bioactive compounds (e.g., quercetin and luteolin) and 126 common target genes were screened. Furthermore, common target genes of CCHs and HCC were mainly enriched in cancer-associated pathways, and six hub genes with statistical significance in survival analysis were selected as key target genes for molecular docking. Additionally, molecular docking showed that the bioactive compounds docked well with the protein receptors of key target genes.

**Conclusion:**

By combining data mining, network pharmacology, molecular docking, and survival analysis methods, we found that CCHs may play a therapeutic role in HCC through regulating the target genes and pathways related to cancer occurrence and development, angiogenesis, metastasis, and prognosis.

## 1. Introduction

As one of the most common cancers, hepatocellular carcinoma (HCC) is characterized by high morbidity and mortality rates. HCC is predicted to be the sixth most frequent cancer and the fourth most common cause of cancer death worldwide in 2018 [1]. In addition, advanced-stage HCC has a 5-year survival rate of only 5–15% [2]. The chief etiologies for HCC include chronic infection with hepatitis B virus (HBV) or hepatitis C virus (HCV), alcoholic liver disease, and nonalcoholic fatty liver disease [3]. Currently, the therapeutic options for patients with HCC are poor and mainly include surgery, liver transplantation, chemoembolization, and molecularly targeted therapy [4, 5]. Moreover, most patients with late-stage HCC have lost the opportunity of surgical resection, and not all patients with advanced HCC are suitable for chemotherapy or targeted therapy. Thus, it is necessary to develop novel approaches for HCC control.

More and more researchers are paying significant interest in traditional Chinese medicine (TCM) which has been used to treat cancer in China for a long time [6]. Furthermore, TCM is effective in improving symptoms, reducing side effects of chemotherapy, suppressing cancer cell growth, and regulating key intracellular signaling pathways [7]. As an important part of TCM, Chinese herbs play a positive role in the management of HCC in China [8]. Previous literature indicated that several TCM prescriptions consisting of Chinese herbs had anti-HCC effects in both basic and clinical research [9, 10]. Moreover, evidence demonstrated that TCM combined with chemotherapy showed significant efficacy and safety in improvement of life quality and reduction of chemotherapy side effects [11]. Nevertheless, TCM prescriptions for treating HCC are often based on the experience of TCM doctors, and it is unclear which core Chinese herbs (CCHs) are effective treatments for HCC. Additionally, the molecular mechanisms underlying the anti-HCC activity of CCHs are not completely clear.

Currently, data mining analysis of TCM prescriptions has become a research focus in the TCM field [12], which can be used to identify CCHs from a large number of clinical prescriptions. In the past, the complicated interactions between “multi-components” and “multi-targets” of Chinese herbs hindered the mechanism study of these herbs. At present, network pharmacology provides an effective solution to overcome these obstacles and can reveal the synergistic effects of complex Chinese herbs on human systems from a holistic view [13]. In the present study, data mining software was applied to determine CCHs for the treatment of HCC, and mechanisms of CCHs on HCC were analyzed by network pharmacology. Besides, survival analysis and molecular docking methods were used to validate the results of network pharmacology analysis.

## 2. Materials and Methods

### 2.1. Data Mining of TCM Prescriptions

Clinical TCM prescriptions intended solely for HCC were collected from studies in the Chinese National Knowledge Infrastructure (CNKI) database. The search was conducted using the following search terms: “(Chinese medicine OR prescription OR decoction) AND (hepatocellular carcinoma OR liver cancer) AND clinical” (date: 1979 to 3 March 2020). The inclusion criteria for the studies included the following: (1) the first diagnosis of cancer patients being HCC; (2) clinical research on oral TCM prescriptions or oral TCM prescriptions combined with Western medicine in the treatment of HCC; (3) experience of TCM experts. Besides, exclusion criteria were as follows: (1) repeated literature and animal experiments; (2) TCM prescriptions in the literature being primarily for treating acute symptoms (e.g., cold and cough); (3) external prescriptions or Chinese patent medicine. Subsequently, eligible studies were screened, and TCM prescriptions were extracted from the included studies. Furthermore, Traditional Chinese Medicine Inheritance Support System (TCMISS, from the Institute of Chinese Materia Medica, China Academy of Chinese Medical Sciences, version 2.5) was employed to identify the CCHs from all prescriptions. TCMISS software has been widely used to analyze TCM prescriptions, and it has the function of text mining, association rules, and complex system entropy clustering methods [12]. In the present study, association rules in TCMISS were utilized to determine CCHs under the condition of support degree ≥126 (20%).

### 2.2. Screening Bioactive Compounds and Target Genes

Bioactive compounds and corresponding targets of CCHs were obtained using Traditional Chinese Medicine Systems Pharmacology Database and Analysis Platform (TCMSP, http://tcmspw.com/tcmsp.php) [14], Integrative Pharmacology-Based Research Platform of TCM (TCMIP, http://www.tcmip.cn/) [15], and Bioinformatics Analysis Tool for Molecular Mechanism of TCM (BATMAN-TCM, http://bionet.ncpsb.org/batman-tcm/) [16]. In this study, all TCM prescriptions composed of Chinese herbs were given orally, and the bioactive compounds were selected under the conditions of drug-likeness (DL) ≥0.18 (mean value for all molecules within the DrugBank database) and oral bioavailability (OB) ≥30% [17]. Additionally, all compounds are numbered using Mol ID from TCMSP, and all target names were converted to official gene names using the UniProt database (https://www.uniprot.org/). Moreover, potential therapeutic target genes associated with HCC were obtained from the MalaCards (https://www.malacards.org/) [18] and OMIM databases (https://omim.org/) [19] using “hepatocellular carcinoma” as the keyword, and known target genes for HCC were screened after removing duplicates.

### 2.3. Constructing the Network of CCHs and Targets

Venn diagrams were used to determine common target genes of CCHs and HCC by “VennDiagram” package in R 3.6.0 (https://www.r-project.org/). Then, the interaction network of CCHs bioactive compounds and common target genes was built using Cytoscape 3.7.1 (https://cytoscape.org/), and the relationships between bioactive compounds of CCHs and common target genes were displayed in the interaction network.

### 2.4. Signaling Pathway and Gene Ontology Analyses

Gene Ontology (GO) and Kyoto Encyclopedia of Genes and Genomics (KEGG) pathway analyses for common target genes were done using the Database for Annotation, Visualization, and Integrated Discovery (DAVID, https://david.ncifcrf.gov/) [20], and false discovery rate (FDR) <0.05 was accepted as significant. In addition, GO analysis was performed according to three categories, namely, biological process (BP), cellular component (CC), and molecular function (MF), and the results were shown as a bar plot using the “ggplot2” package in R 3.6.0.

### 2.5. Protein-Protein Interaction Analysis and Screening for Hub Genes

Protein-protein interaction (PPI) network analysis of the common target genes was completed using Search Tool for the Retrieval of Interacting Genes (STRING) database (https://string-db.org/) [21] with the highest confidence (score >0.9). Then, hub genes were screened from the PPI network by degree, maximum neighborhood component (MNC), and maximal clique centrality (MCC) algorithms in the Cytoscape 3.7.1 plugin, cytoHubba [22, 23]. The overlap genes, predicted by all three algorithms, were selected as hub genes.

### 2.6. Evaluation of Prognostic Values of Hub Genes

The prognostic values of hub genes were assessed by survival analysis using the Kaplan–Meier plotter (http://kmplot.com/analysis/) [24]. The Kaplan–Meier plotter includes data on HCC survival in the Cancer Genome Atlas (TCGA). In survival analysis, overall survival (OS) was analyzed by the Kaplan–Meier (KM) method (log-rank test), and a *p* value less than 0.05 was considered to be a significant difference. In this study, the hub genes that displayed statistically significant differences were considered to be the key target genes.

### 2.7. Molecular Docking of Key Target Proteins and Bioactive Compounds

**Figure 1:**
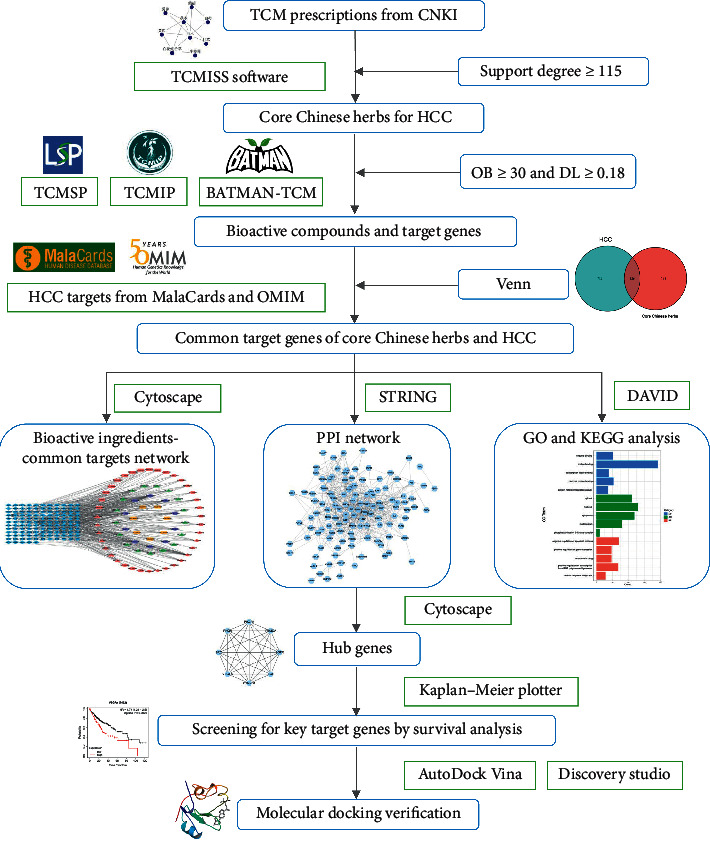
Flowchart of the present study.

In the present study, molecular docking was used to verify the interactions between protein receptors of key target genes and bioactive compounds of CCHs. The corresponding protein receptors of key target genes were acquired using Protein Data Bank (PDB) database (https://www.rcsb.org/) [25], and we included protein receptors based on the following criteria: (1) The structure of protein receptors was determined by X-ray diffraction approach. (2) X-ray resolution <3 Å was preferred. (3) Protein structure with initial ligand was also preferred. AutoDockTools (version 1.5.6, http://autodock.scripps.edu) was utilized to remove the original ligands (if any), excess protein chains, and water molecules of protein receptors [26], and then hydrogens were added to the protein receptors. Furthermore, grid boxes in AutoDockTools were used to identify the docking coordinates. After that, bioactive compounds of CCHs corresponding to key targets were used as ligands, and the “mol2” files of compounds were obtained using TCMSP. Moreover, the files of protein receptors and ligands were converted into “PDBQT” format using AutoDockTools. Finally, AutoDock Vina program (http://vina.scripps.edu/) [27] was used to dock bioactive compounds into the corresponding protein receptors. Besides, docking results were analyzed and visualized using PyMOL (http://www.pymol.org/) and Discovery Studio 2016 (BIOVIA, San Diego, USA). The workflow of our study is displayed in Figure 1.

## 3. Results

### 3.1. Results of Data Mining

**Figure 2:**
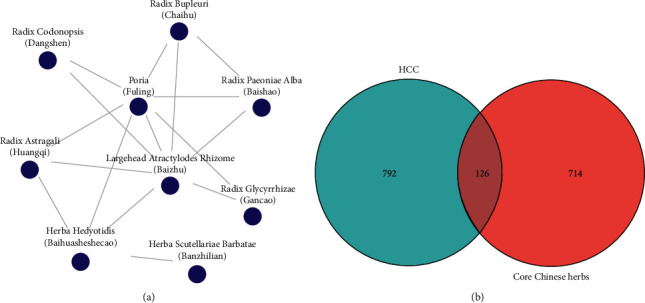
The network of CCHs and the Venn diagram of HCC and CCHs. (a) There are 16 edges and nine nodes in the network. Each edge represents a direct combination of CCHs, and each node represents a core Chinese herb. (b) Venn diagrams showing overlapping target genes between HCC and CCHs.

A total of 1472 studies were identified through CNKI database search. According to the criteria described above, 630 TCM prescriptions for the treatment of HCC were obtained from 527 studies. In addition, the result of TCMISS analysis showed that there were 180 different Chinese herbs used in all prescriptions, and a total of 19 combinations of Chinese herbs that were most commonly used in the TCM prescriptions were found (Table 1). Then, nine CCHs were identified by TCMISS, namely, Largehead Atractylodes Rhizome (Bai Zhu), Poria (Fu Ling), Radix Bupleuri (Chai Hu), Radix Astragali (Huang Qi), Herba Hedyotis (Bai Hua She She Cao), Radix Codonopsis (Dang Shen), Radix Paeoniae Alba (Bai Shao), Radix Glycyrrhizae (Gan Cao), Herba Scutellariae Barbatae (Ban Zhi Lian), which were shown in a network (Figure 2(a)). According to the theory of TCM, Radix Glycyrrhizae (Gan Cao) is often used as a harmonizing (Tiao He) drug, so it was not included in the following analysis. Finally, excluding Radix Glycyrrhizae (Gan Cao), eight CCHs were selected for further analysis.

### 3.2. Bioactive Compounds and Targets

Bioactive compound counts of each core Chinese herb from the three databases are shown in Table 2. After merging the data and removing duplicates, a total of 100 bioactive compounds were identified, and 840 target genes of eight CCHs were identified. Additionally, 191 and 745 HCC-related target genes were obtained from OMIM and MalaCards databases, respectively. Following the removal of duplicates, a total of 918 therapeutic target genes for HCC were found.

### 3.3. Network of Bioactive Compounds and Common Targets

**Figure 3:**
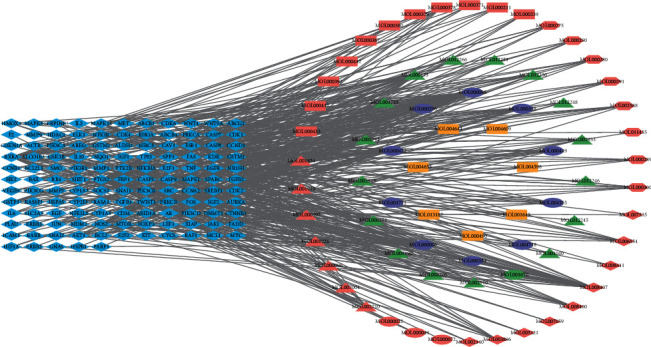
A network of common target genes and CCHs bioactive compounds. In the network, blue diamonds represent the common target genes. Pink, green, and yellow represent the compounds of health-strengthening (Fu Zheng) CCHs, heat-clearing and detoxicating (Qingre Jiedu) CCHs, and relieving liver Qi stagnation (Shu Gan) CCHs, respectively. In the pink shapes, triangles, ellipses, diamonds, hexagons, and rectangles stand for the compounds of Radix Paeoniae Alba, Largehead Atractylodes Rhizome, Radix Codonopsis, Poria, and Radix Astragali, respectively. In green shapes, ellipses and triangles represent the compounds of Herba Hedyotis and Herba Scutellariae Barbatae, respectively. Yellow rectangles represent the compounds of Radix Bupleuri. Purple ellipses indicate common compounds of multiple CCHs.

A total of 126 common target genes of CCHs and HCC were identified using Venn diagram (Figure 2(b)), and the interaction network of CCHs bioactive compounds and common target genes was established, including 198 nodes (72 bioactive compounds and 126 genes) and 510 edges (Figure 3). As shown in Figure 3, eight CCHs were divided into 3 types: health-strengthening (Fu Zheng), heat-clearing and detoxicating (Qingre Jiedu), and relieving liver Qi stagnation (Shu Gan). Furthermore, the top 30 bioactive compounds are presented in Table 3 according to the gene count, and the results showed that quercetin, stigmasta-5,22-dien-3-one, luteolin, wogonin, kaempferol, beta-sitosterol, baicalein, stigmasterol, pyrethrin II, etc. connected with most of the common target genes.

### 3.4. GO and KEGG Analyses Results

**Figure 4:**
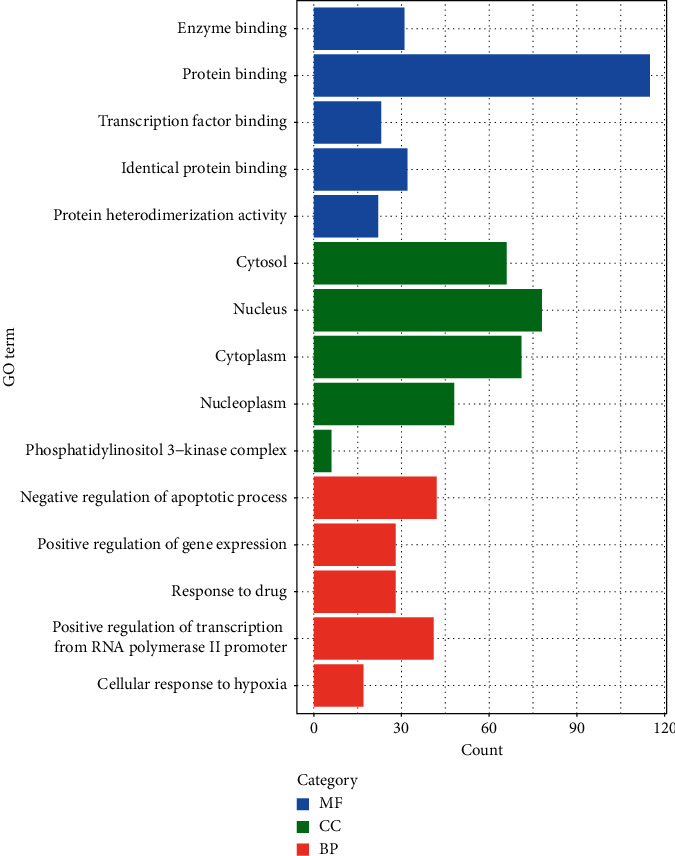
Top five significantly enriched GO terms of each category in GO analysis.

The result of GO analysis revealed that common target genes were mainly enriched in negative regulation of apoptotic process, positive regulation of gene expression, response to drug, positive regulation of transcription from RNA polymerase II promoter, and cellular response to hypoxia (BP); enzyme binding, protein binding, transcription factor binding, identical protein binding, and protein heterodimerization activity (MF); cytosol, nucleus, cytoplasm, nucleoplasm, and phosphatidylinositol 3-kinase complex (CC). Top 5 GO terms of each category are shown in Figure 4. Besides, KEGG pathway analysis demonstrated that most common target genes were related to pathways in cancer, proteoglycans in cancer, hepatitis B, PI3K-Akt, HIF-1 signaling pathway, etc., and the results of the top 20 signaling pathways are listed in Table 4.

### 3.5. PPI Analysis and Hub Genes

**Figure 5:**
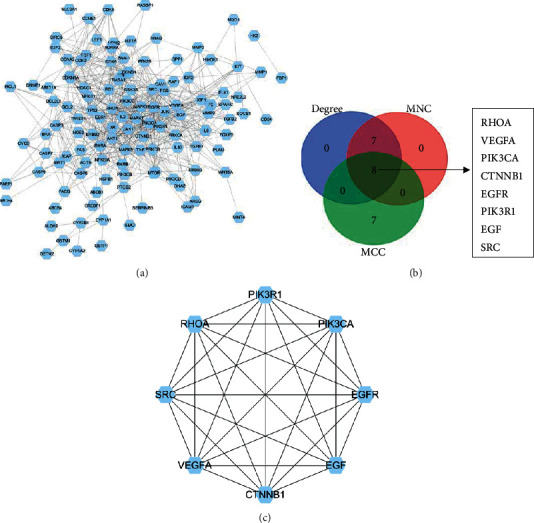
PPI network and hub genes. (a) PPI network of common target genes, consisting of 126 nodes and 698 edges. (b) Top 15 genes were calculated from the PPI network by the degree, MNC, and MCC, respectively. Then, the overlapping genes were screened by Venn diagrams. (c) PPI network of hub genes.

The PPI network with 126 nodes and 698 edges was constructed by STRING and visualized by Cytoscape v. 3.7.1 (Figure 5(a)). Then, hub genes were identified based on MNC, degree, and MCC methods (Figure 5(b)). The interaction network of eight hub genes is shown in Figure 5(c). According to the result of cytoHubba in Cytoscape, the eight hub genes included SRC, PIK3CA, RHOA, PIK3R1, EGFR, VEGFA, EGF, and CTNNB1.

### 3.6. Results of Survival Analysis

**Figure 6:**
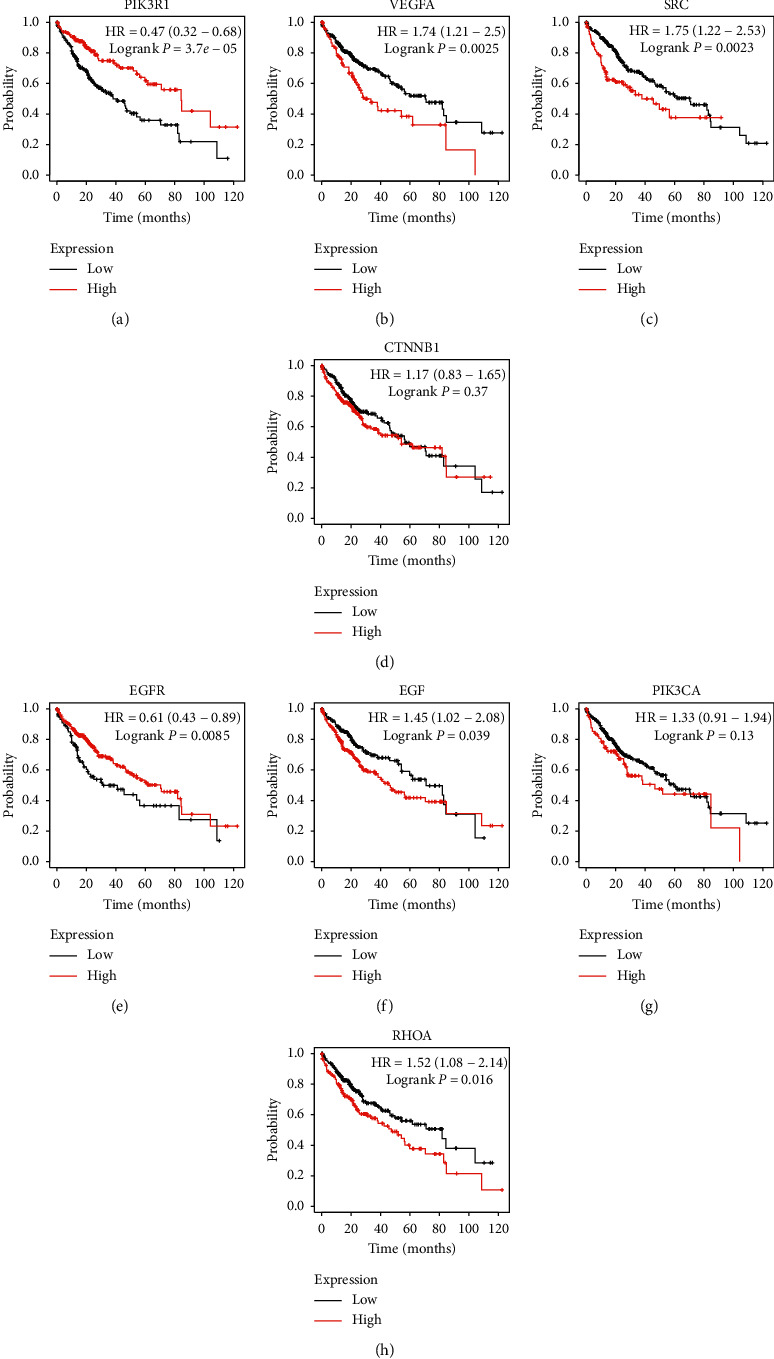
Prognostic values of eight hub genes for OS in patients with HCC. KM survival curves for the hub genes. High expressions of PIK3R1 (a) and EGFR (e) were associated with longer OS in HCC patients, and high expressions of VEGFA (b), SRC (c), EGF (f), and RHOA (h) were correlated with shorter OS. No significant differences were observed in other genes.

KM survival curves showed that high expressions of PIK3R1 and EGFR were correlated with longer OS in patients with HCC, and high expressions of SRC, RHOA, VEGFA, and EGF were associated with shorter OS. Additionally, CTNNB1 and PIK3CA showed no significant differences. The results of survival analysis for the hub genes are presented in Figures 6(a)–6(h). Moreover, six hub genes with statistical significance are defined as key target genes and selected for molecular docking.

### 3.7. Results of Molecular Docking

**Figure 7:**
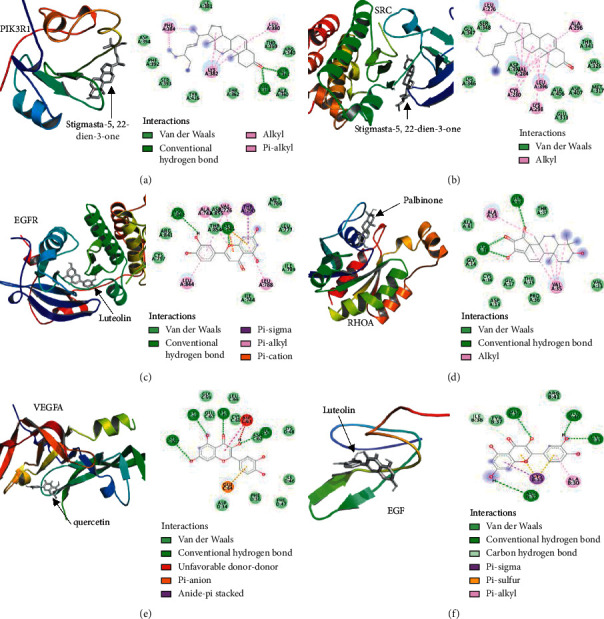
Molecular docking of bioactive compounds with target proteins. (a–f) The first-ranked combinations in each target protein are displayed. The 3D structures of protein receptors of key target genes and ligands (bioactive compounds) are shown in cartoon styles and gray sticks, respectively. The interactions between the protein receptors and compounds of CCHs are displayed in 2D diagrams.

After validating the prognostic values of key genes, protein receptors of key target genes were chosen for molecular docking with corresponding six bioactive compounds of CCHs. The affinity binding values of molecular dockings calculated by AutoDock Vina are demonstrated in Table 5. In the present study, the binding between the receptor (protein receptor of key target gene) and the ligand (bioactive compound) was considered to be good if the affinity value <−5.0 kcal/mol, and the lower the affinity value was, the higher the affinity of the bioactive compound and the protein receptor was. As shown in Table 5, stigmasta-5,22-dien-3-one, luteolin, quercetin, pyrethrin II, palbinone, and baicalein had strong binding affinities for the corresponding proteins, and molecular docking results are shown in Figures 7(a)–7(f).

## 4. Discussion

According to the data mining results, eight Chinese herbs, including Largehead Atractylodes Rhizome, Poria, Radix Bupleuri, Radix Astragali, Herba Hedyotis, Radix Codonopsis, Radix Paeoniae Alba, and Herba Scutellariae Barbatae, were identified as the CCHs for the treatment of HCC. Based on the TCM theory, it is believed that the pathogenesis of HCC is due to “deficiency of healthy Qi,” “liver Qi stagnation,” “heat-toxicity,” etc. In TCM, Largehead Atractylodes Rhizome, Poria, Radix Astragal, Radix Codonopsis, and Radix Paeoniae Alba can be used for “supporting the healthy energy”; Herba Hedyotis and Herba Scutellariae Barbatae have the effect of “clearing heat and removing toxicity”; Radix Bupleuri can be used for “relieving liver Qi stagnation.” Thus, data mining results were highly consistent with the TCM theory. In addition, the bioactive compounds–common targets network showed that the critical bioactive compounds of CCHs could be quercetin, stigmasta-5,22-dien-3-one, luteolin, wogonin, kaempferol, beta-sitosterol, baicalein, Stigmasterol, Pyrethrin II, formononetin, (+)-catechin, and palbinone according to the gene counts (genes ≥10). Previous studies indicated that quercetin and luteolin could inhibit the growth of HCC cells and enhance chemotherapy efficacy [28, 29]. Also, wogonin and kaempferol could effectively suppress the proliferation and invasion of HCC cells by regulating EGFR signaling pathways and PI3K/AKT/mTOR pathway, respectively [30, 31]. Stigmasterol, beta-sitosterol, and baicalein have been found to induce apoptosis of HCC cells by upregulating proapoptotic gene (Bax) and downregulating antiapoptotic gene (Bcl-2) [32–34]. Experimental research showed that formononetin could impede the epithelial-mesenchymal transition (EMT) and malignant progression of HCC [35]. Besides, (+)-catechin could inhibit the proliferation of HCC cells via the caspase-dependent pathway [36]. Overall, previous studies strongly support our findings.

The results of GO analysis showed that common target genes were significantly involved in cellular components and biological processes which are related to cell apoptosis, proliferation, differentiation, and various cellular functions. Moreover, the molecular functions of common target genes may be correlated with the physiological and metabolic processes of liver. KEGG analysis revealed that most of the common target genes were mainly enriched in cancer-related signaling pathways. Previous literature reported that the PI3K/Akt pathway was activated in 30–50% of HCC and the upregulation of p-Akt was correlated with poor survival, metastasis, and vascular invasion in HCC patients [37, 38]. Therefore, PI3K/Akt pathway could shed light on a novel strategy for drug development for HCC. Furthermore, a positive correlation between HBV infection and HCC was observed, and HBV infection can result in the activation of protooncogenes and inactivation of tumor suppressor genes [39, 40]. Proteoglycans are extracellular matrix components of liver microenvironment which play an important role in the progression of HCC and have the potential to be the HCC therapeutic target [41]. It is also reported that kindlin-2 is a member of the focal adhesion protein family which promotes HCC invasion, metastasis [42]. MicroRNA dysregulation has been found to be involved in all stages of HCC, and some microRNAs, such as miR-17-92, miR-21, and miR-221, are generally upregulated in HCC [43]. In addition, HIF-1 plays a critical role in immune escape and EMT of HCC [44]. Literature has shown that the interaction between thyroid hormone and its receptor plays an important role in the regulation of development and proliferation, and metastasis of HCC [45]. Research also showed that the occurrence of HBV-related HCC activates the Ras/MAPK signaling pathway which is correlated with a poor prognosis [46, 47]. Besides, patients with cancer are more likely to suffer from various infections due to low immune function, which may be related to HTLV-I infection, Chagas disease, and Influenza A pathways. Taken together, our results are in line with previous studies, suggesting that CCHs may exert their antitumor effect in HCC by regulating the occurrence, progression, angiogenesis, and metastasis of HCC.

Based on the results of the PPI network and survival curves, we identify six key target genes of CCHs in the treatment of HCC, namely, SRC, RHOA, PIK3R1, EGFR, VEGFA, and EGF. An experiment showed that HBV core protein could promote tumorigenesis of HCC cells by upregulating the expression of SRC protooncogene and then activating SRC/PI3K/Akt pathway [48]. A previous study demonstrated that RHOA (Ras homolog gene family member A) is commonly overexpressed in HCC, and its expression is associated with poor prognosis [49]. Additionally, evidence showed that knockdown of PIK3R1 promoted apoptosis of HCC cells and downregulated p-PI3K and p-AKT expressions in HCC cells [50]. As a growth factor, EGF plays a crucial role in cell proliferation and migration by binding to its receptor EGFR, and high expression of EGF could induce highly malignant HCC [51]. Previous studies suggested that EGFR is overexpressed or mutated in HCC and may be closely related to the formation, invasive growth, and clinical characteristics of HCC [52, 53]. Angiogenesis is closely related to tumor growth and invasion, and HCC is recognized as a typical angiogenic tumor [54]. VEGFA is an inducer of angiogenesis in HCC, and the expression of VEGFA in HCC was significantly higher than that in normal liver tissues [55]. Overall, previous studies made our results more reliable to some extent.

The results of network pharmacology were also confirmed by molecular docking. Table 5 shows that the affinity values of all docking results were less than −5.0 kcal/mol, which indicated that the protein receptors of nine key target genes were docked well with the six different compounds of CCHs. As shown in the three-dimensional mode of Figure 7, six active compounds were successfully docked to the active pocket of protein receptors (SRC, PIK3R1, EGFR, RHOA, VEGFA, EGF), and the two-dimensional diagram in Figure 7 also demonstrates the interactions between active compounds and protein receptors. According to our findings, van der Waals forces, hydrogen bonds, Alkyl, *π*-Alkyl, *π*-Cation, *π*-Sigma, etc. were shown to be involved in the interactions between receptors and compounds. For instance, Figure 7(a) indicates that two hydrogen bonds between stigmasta-5,22-dien-3-one and amino acid residues (ARG-358 and SER-361) of PIK3R1 protein were generated. In addition, the van der Waals force, alkyl, and *π*-alkyl interactions between stigmasta-5,22-dien-3-one and other residues also play an important role. The interpretation of other molecular docking results could use this similar method. These results have successfully validated the network pharmacology data from the perspective of molecular interactions. However, this paper lacks biological experimental confirmation. Both in vivo and in vitro experiments are required to validate our results, and we should take it into consideration in future work. Last but not least, there are few reports about some bioactive compounds (e.g., stigmasta-5,22-dien-3-one, pyrethrin II, palbinone), and further research of these compounds is still needed.

## 5. Conclusions

To sum up, based on a combination of TCM prescription data mining, network pharmacology, KM survival analysis, and molecular docking, we identified eight CCHs for treating HCC and found that CCHs may play a therapeutic role in HCC by regulating the genes and pathways associated with cancer occurrence and development, angiogenesis, metastasis, and prognosis.

## Figures and Tables

**Table 1:** Top 19 commonly used combinations of Chinese herbs according to the association rules.

No.	The combinations of Chinese herbs	Freq.
1	Largehead Atractylodes Rhizome, Poria	284
2	Radix Astragali, Largehead Atractylodes Rhizome	195
3	Radix Codonopsis, Largehead Atractylodes Rhizome	188
4	Radix Codonopsis, Poria	170
5	Largehead Atractylodes Rhizome, Radix Bupleuri	163
6	Radix Bupleuri, Poria	160
7	Radix Astragali, Poria	158
8	Radix Codonopsis, Largehead Atractylodes Rhizome, Poria	158
9	Largehead Atractylodes Rhizome, Radix Paeoniae Alba	145
10	Radix Paeoniae Alba, Radix Bupleuri	145
11	Herba Hedyotis, Largehead Atractylodes Rhizome	142
12	Largehead Atractylodes Rhizome, Radix Glycyrrhizae	141
13	Herba Hedyotis, Poria	140
14	Radix Astragali, Largehead Atractylodes Rhizome, Poria	139
15	Radix Glycyrrhizae, Poria	136
16	Largehead Atractylodes Rhizome, Radix Bupleuri, Poria	135
17	Herba Hedyotis, Radix Astragali	133
18	Radix Paeoniae Alba, Poria	131
19	Herba Hedyotis, Herba Scutellariae Barbatae	126

**Table 2:** Information on bioactive compounds from three TCM databases.

CCHs	TCMSP	TCMIP	BATMAN-TCM	Total
Largehead Atractylodes Rhizome	7	3	1	7
Poria	15	7	6	18
Radix Bupleuri	17	2	10	18
Radix Astragali	20	5	9	25
Herba Hedyotis	7	—	1	7
Radix Codonopsis	21	4	12	24
Radix Paeoniae Alba	13	7	9	18
Herba Scutellariae Barbatae	29	5	5	32

CCHs: core Chinese herbs; TCMSP: Traditional Chinese Medicine Systems Pharmacology Database and Analysis Platform; TCMIP: Integrative Pharmacology-Based Research Platform of TCM; BATMAN-TCM: Bioinformatics Analysis Tool for Molecular Mechanism of TCM.

**Table 3:** Data of the top 30 bioactive compounds in CCHs (gene count ≥6).

Mol ID	Bioactive compounds	OB	DL	Genes	Database
MOL000098	Quercetin	46.43	0.28	58	TCMSP/BATMAN
MOL008407	Stigmasta-5,22-dien-3-one	45.40	0.76	33	TCMSP/TCMIP/BATMAN
MOL000006	Luteolin	36.16	0.25	31	TCMSP
MOL000173	Wogonin	30.68	0.23	26	TCMSP/TCMIP
MOL000422	Kaempferol	41.88	0.24	19	TCMSP/BATMAN
MOL000358	Beta-sitosterol	36.91	0.75	16	TCMSP/BATMAN
MOL002714	Baicalein	33.52	0.21	14	TCMSP
MOL000449	Stigmasterol	43.83	0.76	12	TCMSP/TCMIP/BATMAN
MOL002710	Pyrethrin II	48.36	0.35	12	TCMIP/BATMAN
MOL000392	Formononetin	69.67	0.21	10	TCMSP/BATMAN
MOL000492	(+)-Catechin	54.83	0.24	10	TCMSP/TCMIP/BATMAN
MOL001919	Palbinone	43.56	0.53	10	TCMSP/TCMIP/BATMAN
MOL000378	7-O-Methylisomucronulatol	74.69	0.30	9	TCMSP
MOL000417	Calycosin	47.75	0.24	9	TCMSP/BATMAN
MOL003896	7-Methoxy-2-methyl isoflavone	42.56	0.20	9	TCMSP
MOL004355	Spinasterol	42.98	0.76	9	TCMSP/BATMAN
MOL006554	Taraxerol	38.40	0.77	9	TCMSP/TCMIP/BATMAN
MOL008400	Glycitein	50.48	0.24	9	TCMSP/BATMAN
MOL012250	7-Hydroxy-5,8-dimethoxyflavone	43.72	0.25	9	TCMSP/TCMIP/BATMAN
MOL000300	Dehydroeburicoic acid	44.17	0.83	8	TCMIP/BATMAN
MOL000351	Rhamnazin	47.14	0.34	8	TCMSP
MOL000354	Isorhamnetin	49.60	0.31	8	TCMSP/BATMAN
MOL002588	Eburicol	42.37	0.77	8	BATMAN
MOL002910	Carthamidin	41.15	0.24	8	TCMIP/BATMAN
MOL002933	5,7,4′-Trihydroxy-8-methoxyflavone	36.56	0.27	8	TCMIP/BATMAN
MOL004644	Sainfuran	79.91	0.23	8	BATMAN
MOL008206	Moslosooflavone	44.09	0.25	7	TCMSP
MOL000275	Trametenolic acid	38.71	0.80	6	TCMSP/TCMIP
MOL000280	Dehydrotumulosic acid	31.07	0.82	6	TCMIP
MOL012266	Rivularin	37.94	0.37	6	TCMSP/TCMIP

DL: drug-likeness; OB: oral bioavailability; TCMSP: Traditional Chinese Medicine Systems Pharmacology Database and Analysis Platform; TCMIP: Integrative Pharmacology-Based Research Platform of TCM; BATMAN: Bioinformatics Analysis Tool for Molecular Mechanism of TCM.

**Table 4:** Results of KEGG pathways (top 20).

ID	KEGG pathways	Gene count	FDR
hsa05200	Pathways in cancer	70	1.13*E* − 51
hsa05205	Proteoglycans in cancer	45	1.75*E* − 34
hsa05161	Hepatitis B	44	9.76*E* − 40
hsa04151	PI3K-Akt signaling pathway	36	7.78*E* − 15
hsa05166	HTLV-I infection	34	3.92*E* − 17
hsa05206	MicroRNAs in cancer	32	1.44*E* − 13
hsa04510	Focal adhesion	30	1.17*E* − 15
hsa05215	Prostate cancer	29	1.75*E* − 25
hsa05210	Colorectal cancer	26	1.69*E* − 25
hsa05212	Pancreatic cancer	26	7.14*E* − 25
hsa05222	Small cell lung cancer	26	1.79*E* − 21
hsa04919	Thyroid hormone signaling pathway	26	6.72*E* − 18
hsa05203	Viral carcinogenesis	26	1.37*E* − 11
hsa05223	Non-small-cell lung cancer	24	1.81*E* − 23
hsa05220	Chronic myeloid leukemia	24	1.75*E* − 20
hsa04066	HIF-1 signaling pathway	24	2.71*E* − 17
hsa05142	Chagas disease	24	1.92*E* − 16
hsa05164	Influenza A	24	3.13*E* − 11
hsa04014	Ras signaling pathway	24	8.72*E* − 09
hsa05214	Glioma	23	4.03*E* − 20

FDR: false discovery rate.

**Table 5:** Molecular docking between the target proteins and bioactive compounds of CCHs.

Target	Compound	Affinity (kcal/mol)
PIK3R1	Stigmasta-5,22-dien-3-one	−9.9
SRC	Stigmasta-5,22-dien-3-one	−9.4
EGFR	Luteolin	−8.8
EGFR	Quercetin	−8.7
SRC	Pyrethrin II	−8.2
RHOA	Palbinone	−8.2
VEGFA	Quercetin	−7.8
VEGFA	Luteolin	−7.6
VEGFA	Baicalein	−7.5
EGF	Luteolin	−6.0

## Data Availability

The data that support the findings of our study are openly available in http://tcmspw.com/tcmsp.php, http://www.tcmip.cn/, http://bionet.ncpsb.org/batman-tcm/, https://www.uniprot.org/, https://www.malacards.org/, https://omim.org/, https://david.ncifcrf.gov/, https://string-db.org/, http://kmplot.com/analysis/, and https://www.rcsb.org/. The data used and analyzed during the current study are available from the corresponding author on reasonable request.
